# *Toxoplasma* Cathepsin Protease B and Aspartyl Protease 1 Are Dispensable for Endolysosomal Protein Digestion

**DOI:** 10.1128/mSphere.00869-19

**Published:** 2020-02-12

**Authors:** Christian McDonald, David Smith, Manlio Di Cristina, Geetha Kannan, Zhicheng Dou, Vern B. Carruthers

**Affiliations:** a Department of Microbiology and Immunology, University of Michigan Medical School, Ann Arbor, Michigan, USA; b Department of Chemistry, Biology and Biotechnology, University of Perugia, Perugia, Italy; c Department of Biological Sciences, Clemson University, Clemson, South Carolina, USA; University at Buffalo

**Keywords:** *Toxoplasma gondii*, autophagy, cathepsin, lysosome, proteases

## Abstract

The lysosome-like vacuolar compartment (VAC) is a major site of proteolysis in the intracellular parasite Toxoplasma gondii. Previous studies have shown that genetic ablation of a VAC-residing cysteine protease, cathepsin protease L (CPL), resulted in the accumulation of undigested protein in the VAC and loss of parasite viability during the chronic stage of infection. However, since the maturation of another VAC localizing protease, cathepsin protease B (CPB), is dependent on CPL, it remained unknown whether these defects result directly from ablation of CPL or indirectly from a lack of CPB maturation. Likewise, although a previously described cathepsin D-like aspartyl protease 1 (ASP1) could also play a role in proteolysis, its definitive residence and function in the *Toxoplasma* endolysosomal system were not well defined. Here, we demonstrate that CPB is not necessary for protein turnover in the VAC and that CPB-deficient parasites have normal growth and viability in both the acute and chronic stages of infection. We also show that ASP1 depends on CPL for correct maturation, and it resides in the T. gondii VAC, where, similar to CPB, it plays a dispensable role in protein digestion. Taken together with previous work, our findings suggest that CPL is the dominant protease in a hierarchy of proteolytic enzymes within the VAC. This unusual lack of redundancy for CPL in T. gondii makes it a single exploitable target for disrupting chronic toxoplasmosis.

**IMPORTANCE** Roughly one-third of the human population is chronically infected with the intracellular single-celled parasite Toxoplasma gondii, but little is known about how this organism persists inside people. Previous research suggested that a parasite proteolytic enzyme, termed cathepsin protease L, is important for *Toxoplasma* persistence; however, it remained possible that other associated proteolytic enzymes could also be involved in the long-term survival of the parasite during infection. Here, we show that two proteolytic enzymes associated with cathepsin protease L play dispensable roles and are dependent on cathepsin L to reach maturity, which differs from the corresponding enzymes in humans. These findings establish a divergent hierarchy of proteases and help focus attention principally on cathepsin protease L as a potential target for interrupting *Toxoplasma* chronic infection.

## Abstract (precis)

Roughly one-third of the human population is chronically infected with the intracellular single-celled parasite Toxoplasma gondii, but little is known about how this organism persists inside people. Previous research suggested that a parasite proteolytic enzyme, termed cathepsin protease L, is important for *Toxoplasma* persistence; however, it remained possible that other associated proteolytic enzymes could also be involved in the long-term survival of the parasite during infection. Here, we show that two proteolytic enzymes associated with cathepsin protease L play dispensable roles and are dependent on cathepsin L to reach maturity, which differs from the corresponding enzymes in humans. These findings establish a divergent hierarchy of proteases and help focus attention principally on cathepsin protease L as a potential target for interrupting *Toxoplasma* chronic infection.

## INTRODUCTION

The apicomplexan parasite Toxoplasma gondii is an obligate intracellular protozoan that chronically infects ∼2 billion people worldwide (1). Toxoplasmosis can be fatal for those who are immunocompromised, such as AIDS or organ transplant patients (1, 2). In pregnant women, *Toxoplasma* can overcome barriers at the maternal-fetal interface and parasitize the developing fetus, which can cause a spontaneous abortion or neurological pathologies in the surviving offspring (3). Strikingly, most immunocompetent infected individuals are asymptomatic despite being persistently infected. Insufficient understanding of the underlying pathways that permit long-term infection precludes the development of viable treatment options to limit disease in at-risk individuals.

The endolysosomal pathway is a vital system in eukaryotic organisms. Proteases associated with these systems are of particular interest due to their role in various biological processes, including catabolism and enzyme activation (2, 4, 5). The lysosomal cysteine proteases cathepsins L and B are ubiquitously expressed in eukaryotic cells and together are integral for proteolytic activity within lysosomes (6). Single-knockout experiments in murine models show only a modest or indistinguishable phenotype for cathepsin L or B deficiency, respectively (7, 8). For example, mouse embryonic fibroblasts lacking cathepsin L present a nonlethal deficit in the turnover of autophagosomes (7). This indicates that the absence of one of these proteases can be overcome in mice. However, double-knockout experiments of both CPL and CPB had a profound effect on mouse health, including pronounced neurodegeneration, defects in motility, and, most significantly, lethality 3 weeks after birth (6, 9). In addition, mice deficient in the lysosomal aspartyl protease cathepsin D die within 4 weeks of birth (10). Cathepsin D also regulates the activation of cathepsin B (11). Thus, whereas cathepsins L and B are individually dispensable in mammals, cathepsin D is critical for maintaining proteolytic activity and survival in mammals.

Lysosomal cathepsin proteases are expressed in multicopy families in some pathogenic protozoa, such as Plasmodium falciparum. P. falciparum is one of the causative agents of malaria and, like many other protozoan parasites, relies on proteases for various aspects of its pathogenesis (2). *Plasmodium* obtains host hemoglobin and digests it in a specialized organelle, known as the food vacuole (FV), to procure necessary nutrients (12, 13). Three cathepsin L-like cysteine proteases (falcipains 2a, 2b, and 3) and four cathepsin D-like aspartyl proteases (plasmepsins I, II, and IV and histoaspartic protease) localize to the FV and are key players in hemoglobin degradation (14–16). As with the mammalian systems mentioned above, functional redundancy exists between these proteases (13, 17, 18). Knockout of falcipain-2a in one study resulted in an accumulation of unprocessed hemoglobin in the FV; however, this was a transient phenotype and was subsequently not seen in later stages of the parasite life cycle, presumably due to the expression of falcipains 2b and 3 (17). Likewise, parasites lacking any one of the four plasmepsins were slower growing yet viable (18). Taken together, these findings suggest these proteases work cooperatively in the turnover of host hemoglobin.

Like its *Plasmodium* kin, *Toxoplasma* possesses a recently characterized lysosome-like organelle known as the vacuolar compartment (VAC), which houses proteases canonically associated with lysosomes (19–21). Acute-stage parasites (tachyzoites) have been shown to ingest host cytosolic proteins for the duration of the parasite cell cycle, and the catabolism of these host-derived constituents occurs in the VAC (19, 22). In contrast to *Plasmodium*, *Toxoplasma* encodes single copies of cathepsin L (CPL), cathepsin B (CPB), and cathepsin D (aspartyl protease 1, or ASP1) in its genome. Of the five cathepsin-like cysteine proteases encoded by the *Toxoplasma* genome, the VAC is the major site for CPB and CPL (23–25). Recent investigations of the VAC uncovered how chronic-stage parasites (bradyzoites) sustain themselves in the host indefinitely following encystation. First, the genetic ablation of CPL has shown its contribution to acute-stage virulence and invasion. Second, cystogenic strains of T. gondii deficient in this protease accumulate large quantities of undigested material in the VAC of bradyzoites *in vitro*. Finally, the viability of CPL-deficient bradyzoites was starkly reduced *in vitro*, consistent with vastly reduced cyst burden *in vivo* (26). It remains unclear whether this is due solely to CPL disruption or due to CPB maturation being dependent on CPL (23). In other words, it is unknown whether the phenotypes observed in CPL-deficient parasites are due to a lack of active CPB.

Of the seven aspartyl proteases identified in the *Toxoplasma* genome, only one, ASP1, shares a clade with the P. falciparum FV plasmepsins (27, 28). This phylogenetic relationship may point to a digestive function within the T. gondii endolysosomal system, including the VAC. In interphase parasites, ASP1 was reported to localize to vesicular structures in the parasite apical region that are distinct from apical secretory organelles (micronemes, rhoptries, and dense granules) (27). During daughter cell formation, ASP1 redistributed to the inner membrane complex (IMC) that delineates developing daughter parasites. Thus, although these findings suggest a dynamic localization of ASP1, the extent to which its phylogenetic heritage aligns with the occupation of an endolysosomal compartment remains unknown.

Although a critical requirement for protein turnover in the VAC in chronic-stage T. gondii was shown previously, key questions remain regarding the contribution of specific proteases to this degradation system. Therefore, in this study our aim was to define the role of T. gondii CPB and ASP1 in protein turnover in the VAC, delineate their position within a protease hierarchy, and determine their contribution to infection.

## RESULTS

### Determining the extent of CPB’s role in tachyzoite growth and host-derived protein turnover.

**FIG 1:**
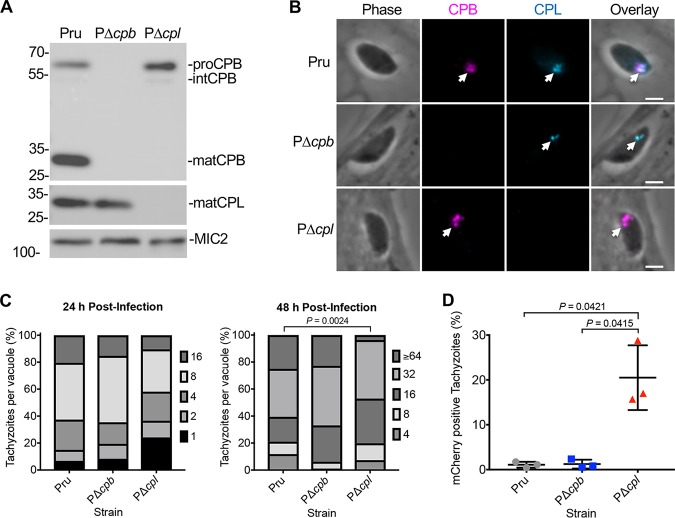
CPB does not play a major role in tachyzoite growth and protein turnover. (A) Immunoblot of tachyzoite lysates probed for T. gondii CPL and CPB along with MIC2 as a loading control. Protein bands are labeled as proform (pro), intermediate form (int), or mature form (mat) based on their molecular weights. (B) Recently invaded intracellular tachyzoites were stained with anti-CPB (violet) and anti-CPL (blue). Arrowheads indicate labeling of the VAC. Scale bars, 2 μm. (C) T. gondii tachyzoites were cultured in HFF monolayers. Samples were collected at 24 and 48 h postinfection, fixed, and stained with anti-SAG1 antibody, and the number of parasites per vacuole was quantified. Percentages of various replication stages for each strain are quantified. Results represent means from 2 or 3 biological replicates. The total numbers of parasitophorous vacuoles counted across 3 biological replicates were the following: Pru, 147 and 152; PΔ*cpb*, 144 and 145; PΔ*cpl*, 153 and 136 (values are for 24 and 48 h postinfection, respectively). Only statistically significant differences are represented. Statistical significance was determined by unpaired two-tailed Student’s *t* test performed on the mean number of parasites per vacuole for each strain across 3 biological replicates. (D) Assay quantification of tachyzoite ingestion of mCherry showing means ± standard deviations from 3 biological replicates. The data generated were analyzed using the following number of tachyzoites per replicate: Pru, *n =* 469, 464, and 231; PΔ*cpb*, *n =* 374, 613, and 213; and PΔ*cpl*, *n =* 427, 299, and 216. All strains were compared, and only significant differences are shown. Unpaired two-tailed *t* test with Welch’s corrections was performed on the means from 3 biological replicates.

Maturation of the *Toxoplasma* protease CPB was shown to be dependent on another VAC-residing protease, CPL. Since CPL-deficient parasites exhibit numerous phenotypes and do not produce mature CPB, it is unclear whether the phenotypes described are attributed to inactive CPB. To assess the role of CPB in T. gondii, we knocked out CPB in the cystogenic type II Prugniaud Δ*ku80* strain (29) (termed Pru here) and confirmed this by PCR analysis (see Fig. S1 in the supplemental material), immunoblotting, and immunofluorescence (Fig. 1A and B). The inclusion of the previously described PΔ*cpl* strain (26) in the immunoblot confirmed that maturation of CPB is dependent on expression of CPL, which is consistent with previous findings in a type I strain (23). Similarly, we found that CPB localizes to the VAC of tachyzoites (Fig. 1B), which is also consistent among strains (23). To test how tachyzoite replication was affected following CPB knockout, Pru, PΔ*cpb*, and PΔ*cpl* tachyzoites were grown in human foreskin fibroblast (HFF) cells, followed by the enumeration of parasites per parasitophorous vacuole (PV) to quantify replication. At 24 h postinfection, no measurable differences were seen between these strains; however, at 48 h postinfection, PΔ*cpl* tachyzoites had slower growth than Pru, whereas replication of PΔ*cpb* was indistinguishable from that of Pru (Fig. 1C). As mentioned above, the ingestion of host-derived material has been shown in the VAC of acute-stage parasites. Using a Chinese hamster ovarian (CHO)-K1 cell line inducibly expressing mCherry, we found that the accumulation of host-derived mCherry was significantly higher in PΔ*cpl* tachyzoites than in both Pru and PΔ*cpb* strains, whereas PΔ*cpb* parasites showed accumulation comparable to that of Pru (Fig. 1D). In all, these findings suggest that CPB has a limited role in tachyzoite VAC digestive function.

10.1128/mSphere.00869-19.1 FIG S1 Creation and validation of PΔ*cpb* parasites. (A) Schematic diagram of double-crossover homologous replacement of *CPB* with a dihydrofolate dehydrogenase (DHFR) selectable marker. The vector used to delete *CPB* was generated with the following steps: 1-kb region of the 5′-UTR of the *CPB* gene was PCR amplified with ApaI and HindIII engineered at its 5′ and 3′ ends, respectively, and cloned using these sites in the pMDC64 plasmid upstream of the DHFR cassette carried by this vector. A 1-kb region of the 3′-UTR of the *CPB* gene then was amplified by PCR with BamHI and SacII sites incorporated at its 5′ and 3′ ends, respectively, and cloned downstream of the *DHFR* resistance cassette. The resulting final plasmid was digested with the enzymes ApaI and SacII to release the backbone from the repair template and introduced into PruΔ*ku80* parasites by electroporation. The transfected parasites were subjected to 2 μM pyrimethamine selection. Clones of the *CPB*-deficient parasites were isolated by limiting dilution. (B) Replacement of the *CPB* gene with DHFR was confirmed by PCR. (C) Primers used to generate the *CPB* knockout vector to test either the integration of the DHFR cassette in the *CPB* locus or the absence of the *CPB* gene. Download FIG S1, TIF file, 0.5 MB. Copyright © 2020 McDonald et al. 2020 McDonald et al. This content is distributed under the terms of the Creative Commons Attribution 4.0 International license.

### CPB maturation depends on CPL activity in bradyzoites.

**FIG 2:**
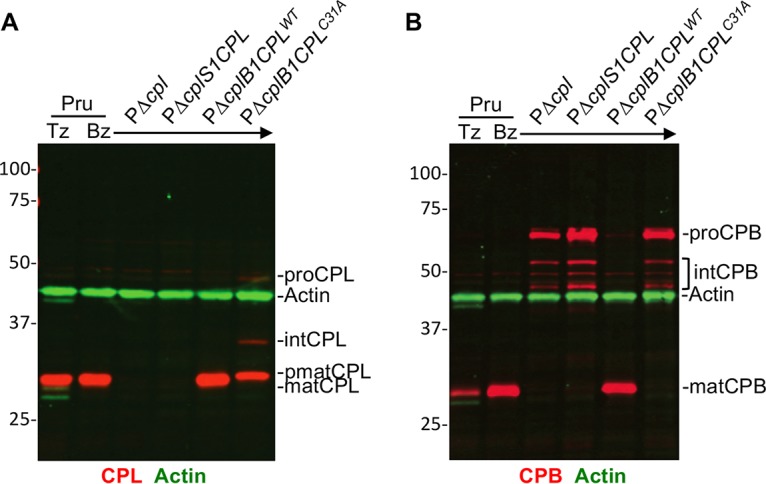
T. gondii CPB maturation is dependent on the expression of active CPL. Li-COR Western blots of tachyzoite and bradyzoite cell lysates probed for T. gondii CPL (red in panel A), CPB (red in panel B), and actin (green in panels A and B) as the loading control. Protein bands are labeled as proform (pro), intermediate form (int), pseudomature form (pmat), and mature form (mat) based on their molecular weights.

We used a previously described series of strains (26) to explore the role of CPL activity in the maturation of itself and CPB in chronic-stage bradyzoites. PΔ*cplS1CPL* and PΔ*cplB1CPL* express CPL exclusively in tachyzoites and bradyzoites, respectively. PΔcplB1CPL* exclusively expresses a catalytically inactive form of CPL in bradyzoites. Using immunoblotting, we found CPL catalytic activity is necessary for normal self-maturation based on observing a pseudomature (pmatCPL) species in PΔcplB1CPL* bradyzoites (Fig. 2A). These results are consistent with previous findings in tachyzoites (23). When probed for CPB, immunoblots also displayed bands for mature CPB only in strains that express active CPL, namely, Pru and PΔ*cplB1CPL* bradyzoites (Fig. 2B). Immature CPB was observed in ΔcplB1CPL*, confirming that CPL activity is necessary for maturation of CPB in bradyzoites.

### CPB is not necessary for turnover of autophagic material in bradyzoites.

**FIG 3:**
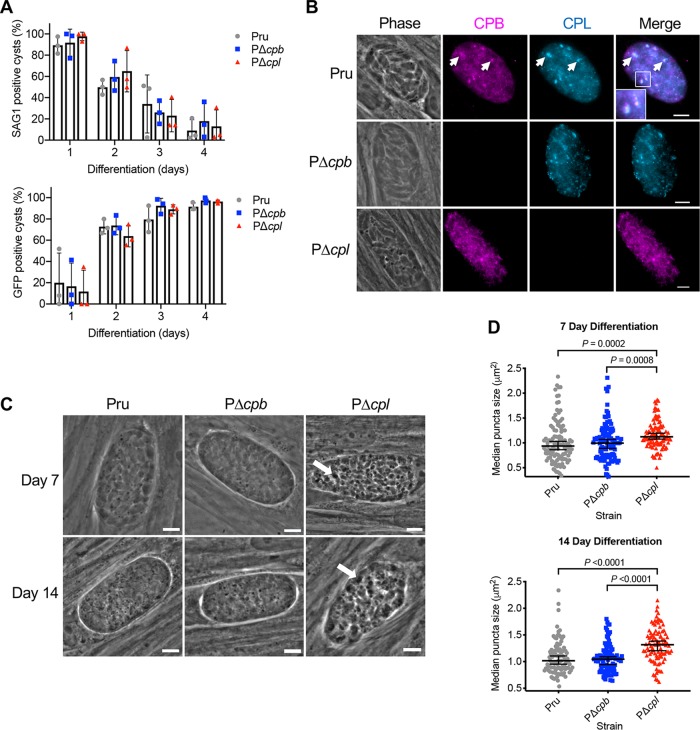
CPL, not CPB, is the major protease necessary for the turnover of autophagosomes in the VAC in bradyzoites. (A) The rate of parasite differentiation from the tachyzoite stage to the bradyzoite stage was determined *in vitro* by assessment of the tachyzoite-specific antigen SAG1 and GFP under the control of a bradyzoite LDH2 promoter. Infected monolayers were cultured for the indicated days, fixed, probed for SAG1, and quantified. Bars indicate means ± standard deviations from 3 biological replicates. Experiments analyzed 105, 106, 153, and 151 cysts for Pru, 117, 123, 149, and 110 cysts for PΔ*cpb*, and 75, 108, 155, and 130 cysts for PΔ*cpl* on days 1, 2, 3, and 4, respectively. All strains were compared, and only statistical significance is shown. Unpaired two-tailed *t* test with Welch’s corrections was performed on the means from 3 biological replicates. (B) Immunofluorescence localization of CPB and CPL in bradyzoites. Parasite strains were differentiated for 7 days, fixed, and stained with anti-CPB (violet) and anti-CPL (blue). The inset in the merged image of Pru shows enlargement of the boxed region. Scale bars, 5 μm. (C) Phase-contrast microscopy was used to image bradyzoites *in vitro* following 7- and 14-day cultures. Enlarged dark puncta seen in PΔ*cpl* bradyzoites are indicative of defective protein degradation in the VAC (arrows). Scale bars, 5 μm. (D) The size of the puncta in bradyzoites was measured. The following numbers of cysts across 3 biological replicates were used to analyze each strain: Pru (day 7, 109 cysts; day 14, 98 cysts), PΔ*cpb* (day 7, 106 cysts; day 14, 102 cysts), and PΔ*cpl* (day 7, 105 cysts; day 14, 97 cysts). Lines represent medians with 95% confidence intervals. For this data set, ROUT with a *Q* value of 0.1% was used to identify and remove 1 outlier each for Pru and PΔ*cpl* for 14 days postdifferentiation. Since the data did not fit a normal distribution, a nonparametric Kruskal-Wallis test with Dunn’s multiple comparisons was used to compare the medians of data combined from 3 biological replicates. All strains were compared, and only significant differences are shown.

We have previously demonstrated that CPL deficiency in T. gondii leads to the accumulation of autophagic material in the VAC in bradyzoites (26). However, due to CPB maturation being dependent on the proteolytic activity of CPL, parasites deficient in CPL will also be deficient in active CPB. Therefore, it remained a possibility that this accumulation was actually due to a lack of active CPB in the VAC. To address this, we first used surface antigen 1 (SAG1) as a tachyzoite stage-specific marker as well as green fluorescent protein (GFP), which is expressed following bradyzoite differentiation (29), and found that differentiation was similar among the Pru, PΔ*cpb*, and PΔ*cpl* strains (Fig. 3A). Consistent with our observations of CPB and CPL colocalization in tachyzoites, these two proteases were also found to colocalize in bradyzoite cysts (Fig. 3B). To determine the extent to which CPB independently contributed to the turnover of autophagic material, we assessed the accumulation of dense puncta, indicative of undigested autophagosomes in PΔ*cpb* bradyzoites, and compared this accumulation to that of Pru and PΔ*cpl* bradyzoites. Our results showed that after 7 and 14 days of differentiation, punctate structures were larger in PΔ*cpl* bradyzoites than Pru ones (Fig. 3C and D). However, no difference in puncta size was seen in PΔ*cpb* bradyzoites compared to Pru bradyzoites. Collectively, our findings suggest that CPB does not play a major digestive role in the endolysosomal system of *Toxoplasma* during acute or chronic infection *in vitro*.

### ASP1 resides in the *Toxoplasma* VAC and is dependent on CPL for normal maturation.

**FIG 4:**
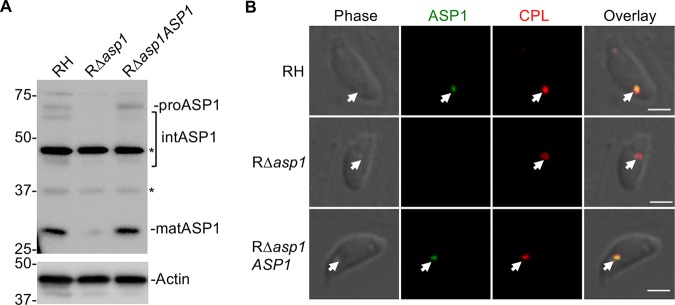
T. gondii ASP1, a cathepsin D ortholog that localizes to the VAC. (A) Immunoblot detection of T. gondii ASP1 in tachyzoites. Protein bands are labeled as proform (pro), intermediate form (int), and mature form (mat) based on their molecular weights. Polypeptides marked with asterisks are nonspecific bands. (B) Immunofluorescence assay of tachyzoites after fixation, staining, and probing for T. gondii ASP1 (green) and CPL (red). Arrowheads indicate colocalization of ASP1 with CPL in the VAC. Scale bars, 2 μm.

Unlike the related apicomplexan, *Plasmodium*, *Toxoplasma* possesses one group A aspartyl protease, ASP1 (27). T. gondii ASP1 was shown to have punctate localization that extensively changes during tachyzoite replication in daughter cells and was predicted to be compartmentalized (27). The *Toxoplasma* VAC displays similar fragmentation during replication (21); therefore, we hypothesized that ASP1 resides within the VAC. To address this definitively, we generated antibodies to full-length recombinant ASP1 and created an ASP1-deficient mutant in the RH strain background (RΔ*asp1*) along with genetically complementing the mutant (RΔ*asp1ASP1*) (Fig. S2). Immunoblots displayed mature ASP1 in parental strain RH and RΔ*asp1ASP1* complement strain but not in RΔ*asp1* (Fig. 4A). Note that despite affinity purifying the antibody on recombinant ASP1, several nonspecific bands were observed in the RΔ*asp1* lysate, including one that nearly comigrates with mature ASP1. PCR analysis (Fig. S2) and immunofluorescence staining confirmed the absence of ASP1 expression in newly invaded RΔ*asp1* tachyzoites. Immunofluorescence staining also revealed colocalization of ASP1 with CPL in RH and RΔ*asp1ASP1* (Fig. 4B).

10.1128/mSphere.00869-19.2 FIG S2 Creation and validation of Δ*asp1* and Δ*asp1ASP1* strains. To generate the ASP1 deletion mutant, a 3-kb region of the 5′-UTR of the *ASP1* gene was PCR amplified with KpnI and ApaI sites engineered at its 5′ and 3′ ends, respectively. The resulting PCR product was digested by KpnI and ApaI and cloned in a plasmid upstream of the bleomycin resistance cassette (BLE). A 3-kb region of the 3′-UTR of the *ASP1* gene next was amplified by PCR with SpeI and XbaI sites incorporated at its 5′ and 3′ ends, respectively, and cloned downstream of the *BLE* resistance cassette. To distinguish random integration of the *BLE* cassette into the parasite genome, a GFP expression cassette was PCR amplified with XbaI sites engineered at both ends and cloned downstream from the 3′-UTR region of *ASP1*. (A) The resulting final plasmid was introduced into wild-type RH parasites by electroporation. The transfected parasites were subjected to bleomycin selection at 50 μg/ml twice during their extracellular stage. Clones of the *ASP1*-deficient parasites were isolated by limiting dilution. To complement the Δ*asp1* mutant, we PCR amplified the coding sequence of *ASP1* from the *Toxoplasma* cDNA library and ∼1 kb of 5′- and 3′-UTRs of *ASP1* from *Toxoplasma* genomic DNA. All three PCR fragments were gel purified and assembled into the pMDC64 plasmid, which encodes a pyrimethamine resistance cassette, by Gibson Assembly. The resulting *ASP1* complementation construct was electroporated into the Δ*asp1* parasites, followed by pyrimethamine selection and limited dilution to isolate single clones. (B) Removal of the *ASP1* gene by BLE cassette was confirmed by PCR. (C) Primers used to generate the *ASP1* knockout vector to test either the integration of the BLE cassette in the *ASP1* locus or the absence of the *ASP1* gene. Download FIG S2, TIF file, 0.3 MB. Copyright © 2020 McDonald et al. 2020 McDonald et al. This content is distributed under the terms of the Creative Commons Attribution 4.0 International license.

**FIG 5:**
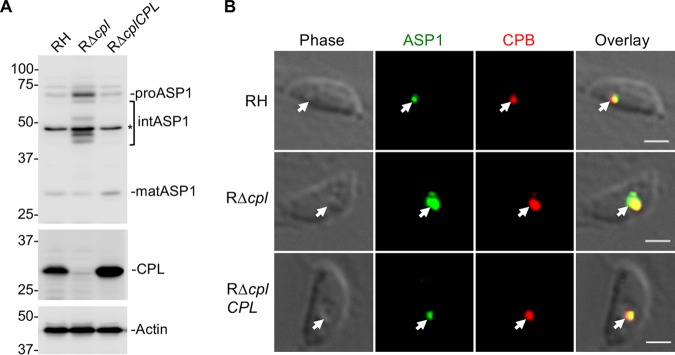
T. gondii immature ASP1 accumulates in tachyzoites lacking active CPL in the VAC. (A) Immunoblot of tachyzoite lysates probed for ASP1 and CPL along with actin as a loading control. For ASP1, based on molecular weights, protein bands are labeled as proform (pro), intermediate form (int), and mature form (mat). (B) Immunofluorescence assay to detect T. gondii ASP1 (green) and CPB (red) in RH, RΔ*cpl*, and RΔ*cplCPL* tachyzoites. Arrowheads show localization of ASP1 with CPB in the VAC. Scale bars, 2 μm.

Since CPL expression is required for maturation of CPB, we reasoned that CPL might also contribute to the maturation of ASP1. Accordingly, immunoblotting of RH strain parasites lacking CPL (RΔ*cpl*) with anti-ASP1 revealed the accumulation of proASP1 and intermediate species of ASP1 (Fig. 5A), suggesting a role for CPL expression in ASP1 maturation. Note that the nonspecific band nearly comigrating with mature ASP1 confounded interpretation of how much mature ASP1 is present in RΔ*cpl* tachyzoites. We also observed a corresponding increase in the staining for ASP1 and CPB in RΔ*cpl* tachyzoites (Fig. 5B), indicating the accumulation of immature species of these proteases in the absence of CPL expression. Collectively, our findings suggest that ASP1 resides in the VAC and that its maturation involves CPL. The findings do not, however, rule out the possibility of other proteases contributing to the maturation of ASP1.

### ASP1 is not required for tachyzoite replication or digestion of host-derived protein in the VAC.

**FIG 6:**
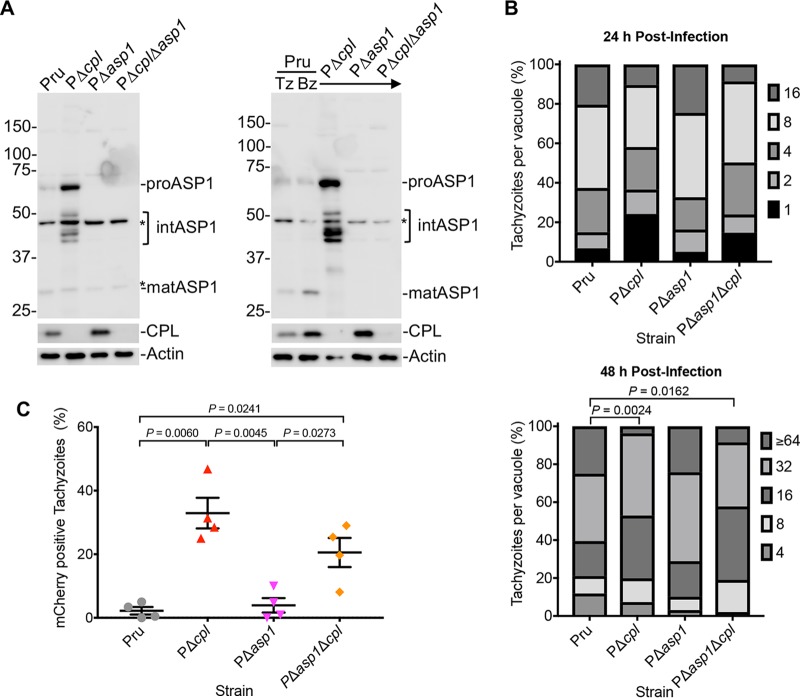
ASP1 is dispensable for tachyzoite replication or ingestion. (A) Parasite lysates of tachyzoites and bradyzoites were immunoblotted with antibodies against T. gondii ASP1 and CPL along with actin as a loading control. For ASP1, based on molecular weights, protein bands are labeled as proform (pro), intermediate form (int), and mature form (mat). (B) T. gondii tachyzoites were cultured in HFF monolayers. Samples were collected at 24 and 48 h postinfection, fixed, and stained with anti-SAG1 antibody, and the numbers of parasites per vacuole were quantified. Percentages of various replication stages for each strain are quantified. The total number of parasitophorous vacuoles counted across 2 biological replicates was the following: Pru, 147 and 152; PΔ*cpl*, 153 and 136; PΔ*asp1*, 159 and 128; and PΔ*asp1*Δ*cpl*, 163 and 142 (all values are for 24 and 48 h postinfection, respectively). Only statistically significant differences are represented. Statistical significance was determined by unpaired two-tailed Student’s *t* test performed on the mean number of parasites per vacuole for each strain across 3 biological replicates. (C) Assay quantitation of tachyzoite ingestion of mCherry showing means ± standard deviations from 4 biological replicates. The data generated were analyzed using the following number of tachyzoites per replicate: Pru (*n =* 205, 219, 223, and 207), PΔ*cpl* (*n =* 224, 242, 224, and 223), PΔ*asp1* (*n =* 218, 220, 220, and 210), and PΔ*asp1*Δ*cpl* (*n =* 203, 235, 228, and 246). All strains were compared, and only significant differences are shown. Unpaired two-tailed *t* test with Welch’s corrections was performed to determine statistical significance.

To further investigate the maturation and function of ASP1 in infection, we generated Pru parasites lacking ASP1 alone (PΔ*asp1*) or lacking ASP1 and CPL (PΔ*asp1*Δ*cpl*) (Fig. S3). Immunoblotting of tachyzoite (Fig. 6A, left) or bradyzoite (Fig. 6A, right) corroborated a role for CPL in ASP1 maturation. Because the nonspecific band that comigrated with mature ASP1 is absent from bradyzoites, it also is apparent that maturation of ASP1 is strongly dependent on the expression of CPL in bradyzoites. The increased expression of both ASP1 and CPL in bradyzoites compared to that of tachyzoites is also evident (Fig. 6A, right). To assess the role of ASP1 in tachyzoite replication, parasites were allowed to infect HFF monolayers for 24 and 48 h. Enumeration of the parasites per PV displayed significant differences only after 48 h postinfection in PΔ*cpl* and PΔ*asp1*Δ*cpl* strains (Fig. 6B). Interestingly, PΔ*asp1*Δ*cpl* parasites did not replicate more slowly than Δ*cpl* parasites, implying that the absence of CPL is principally responsible for the replication phenotype. We next tested for a possible role for ASP1 in digestion of host-derived protein in the VAC. Following the incubation of parasites with mCherry-expressing CHO-K1 cells, PΔ*asp1* failed to accumulate host-derived mCherry, whereas accumulation was seen in PΔ*cpl* and PΔ*cpl*Δ*asp1* tachyzoites (Fig. 6C). These results mirror what was seen in CPB and indicate that neither CPB nor ASP1 is a major contributor to tachyzoite growth or endolysosomal digestive function, whereas CPL has a dominant role.

10.1128/mSphere.00869-19.3 FIG S3 Creation and validation of PΔ*asp1* and PΔ*cpl*Δ*asp1* strains. (A) Schematic diagrams of homologous replacement of *ASP11* with either the DHFR or BLE selectable marker in PruΔ*ku80* and PΔ*cpl*, respectively. The repair template used to obtain *ASP1* deletion was generated with the following procedure: in a first run of PCR, three DNA fragments encompassing a 1-kb region of the ASP1 5′-UTR, the selectable marker, and the *ASP1* 3′-UTR were generated. The selectable marker was amplified using a forward and reverse primer carrying 40 bp complementary to the ASP1 5′- and 3′-UTR, respectively, to allow the fusion of the 3 fragments during a second round of PCR using the forward and reverse primer binding to the ASP1 5′- and 3′-UTR, respectively. Five milligrams of the fusion PCR product was introduced into either PruΔ*ku80* or PΔ*cpl* parasites by electroporation. The transfected parasites were subjected to drug selection, and *ASP1*-deficient clones were isolated by limiting dilution. Deletion of the *ASP1* gene was confirmed by PCR in PruΔ*asp1* (B) and PΔ*cplΔasp1* (C). (D) Primers used to generate the *asp1* KO repair template and to test either the integration of a selectable cassette in the *asp1* locus or the absence of the *asp1* gene. (E) Immunofluorescence assay of tachyzoites after fixation, staining, and probing for T. gondii ASP1 (green) and CPL (red). Scale bars, 2 μm. Download FIG S3, TIF file, 1.0 MB. Copyright © 2020 McDonald et al. 2020 McDonald et al. This content is distributed under the terms of the Creative Commons Attribution 4.0 International license.

### ASP1 plays a dispensable role in proteolytic turnover of autophagic material.

**FIG 7:**
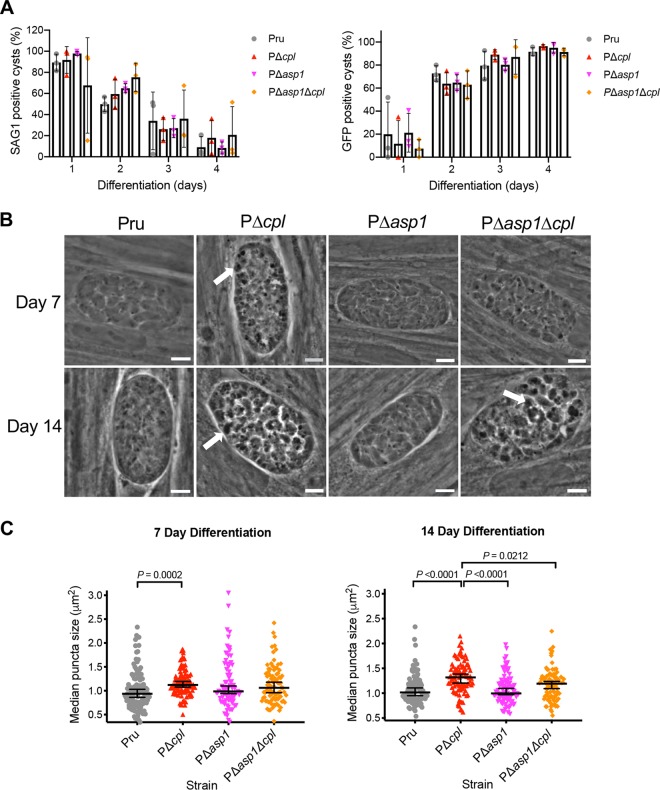
T. gondii ASP1 has a limited role in protein turnover of autophagosomes. (A) The rate of parasite differentiation from the tachyzoite stage to the bradyzoite stage was determined *in vitro* by assessment of the tachyzoite-specific antigen SAG1 and GFP under the control of a bradyzoite LDH2 promoter. Infected monolayers were cultured for the indicated days, fixed, probed for SAG1, and quantified. Bars indicate means ± standard deviations from 3 biological replicates. Experiments analyzed 105, 106, 153, and 151 cysts for Pru, 75, 108, 155, and 130 cysts for PΔ*cpl*, 119, 124, 189, and 146 cysts for PΔ*asp1* and 78, 110, 112, and 161 cysts for PΔ*asp1*Δ*cpl* on days 1, 2, 3, and 4, respectively. All strains were compared, and only statistical significance is shown. Unpaired two-tailed *t* test with Welch’s corrections was performed on the means across 3 biological replicates. (B) Phase-contrast microscopy was used to image bradyzoites *in vitro* following 7- and 14-day culture under differentiation conditions. Enlarged dark puncta indicative of defective protein degradation in VACs are visible in PΔ*cpl* bradyzoites and PΔ*asp1*Δ*cpl* cysts (arrows). Scale bars, 5 μm. (C) The size of the punctae in bradyzoites was measured. The following numbers of cysts across 3 biological replicates were used to analyze each strain: Pru (day 7, 109 cysts; day 14, 98 cysts), PΔ*cpl* (day 7, 105 cysts; day 14, 97 cysts), PΔ*asp1* (day 7, 85 cysts; day 14, 97 cysts), PΔ*asp1*Δ*cpl* (day 7, 90 cysts; day 14, 97 cysts). Lines represent medians with 95% confidence intervals. For this data set, ROUT with a *Q* value of 0.1% was used to identify and remove 1 outlier from PΔ*asp1* for 7 days postdifferentiation and 1 outlier each for Pru and PΔ*cpl* for 14 days postdifferentiation. Since the data did not fit a normal distribution, we used a nonparametric Kruskal-Wallis test with Dunn’s multiple comparisons to compare the medians of data combined from 3 biological replicates. All strains were compared, and only significant differences are shown.

It was also important to establish whether ASP1 could have a functional overlap in the turnover of autophagic material in bradyzoites. Therefore, similar to procedures for PΔ*cpb* parasites, we compared the differentiation and size of puncta in PΔ*asp1* and PΔ*asp1*Δ*cpl* bradyzoites to those of Pru and PΔ*cpl* bradyzoites after 7 and 14 days of differentiation. Differentiation was indistinguishable among the strains based on the decrease in SAG1-positive vacuoles and the corresponding increase of GFP-positive cysts (Fig. 7A). Automated measurement of puncta size also indicated no difference between PΔ*asp1* and Pru bradyzoites in turnover of autophagosomes at day 7 or 14 of differentiation, which is distinct from the enlarged puncta observed in PΔ*cpl* (Fig. 7B and C). Interestingly, PΔ*asp1*Δ*cpl* bradyzoites showed an intermediate phenotype, with puncta that were smaller than those of PΔ*cpl* but potentially larger than those of Pru. Although the basis for this intermediate phenotype remains to be determined, the findings indicate that turnover of autophagosomes is not exacerbated by deletion of both ASP1 and CPL. Taken together, our results suggest that, similar to CPB, ASP1 does not play a major role in the proteolytic turnover of autophagic material in the VAC in bradyzoites, whereas CPL has a dominant role in this activity.

### CPL plays a principal role in bradyzoite viability.

**FIG 8:**
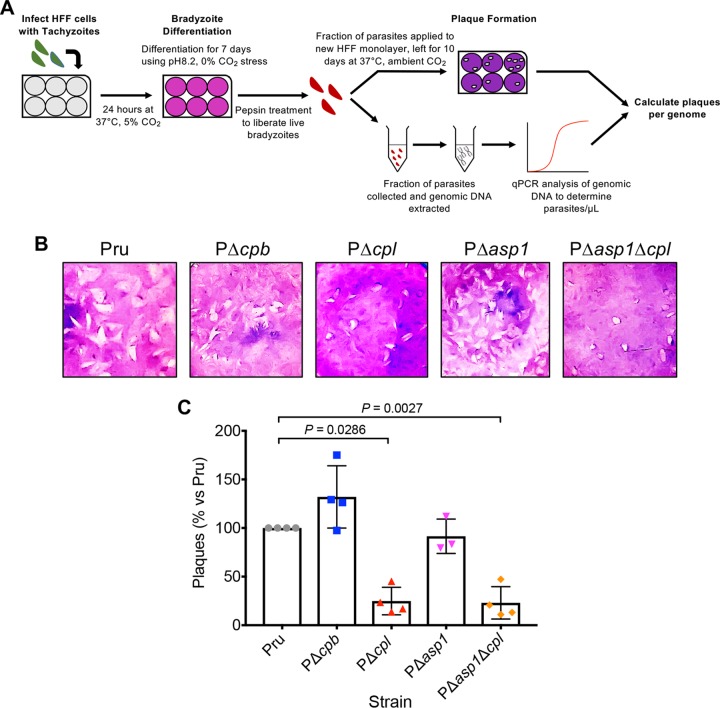
CPL is a central protease in bradyzoite viability. (A) Schematic illustration of the workflow used to examine bradyzoite viability. Experiments conducted were used to generate the data shown in panels B and C. Details are provided in Materials and Methods. (B) Plaque assays were performed on bradyzoites to determine the viability of each strain, based on their ability to undergo the lytic cycle following forced encystation. Plaque zones were visualized following crystal violet staining of the HFF monolayer. (C) Quantitation of the plaque assay described for panel B. Quantitative PCR analysis was used to determine the number of parasites applied to fresh HFF monolayers for the bradyzoite plaque assay, allowing for the number of plaques per genome to be calculated. Data were normalized to those for the parental strain (Pru) and are shown as a percentage. Bars indicate means ± standard deviations from 3 or 4 biological replicates. Only significant differences between Pru and subsequent strains are indicated. Mann-Whitney test was used to assess statistical significance.

To quantify the extent to which CPL, CPB, and ASP1 contribute to bradyzoite viability, we measured the ability of bradyzoites lacking these proteases to invade, differentiate to tachyzoites, and replicate within HFF monolayers, which, if left undisturbed, results in the formation of plaques (Fig. 8A). Because proteolytic deficiency in the VAC is associated with accumulation of puncta and no increases in puncta were observed for PΔ*cpb* or PΔ*asp1* between 7 and 14 days of differentiation, we only measured bradyzoite viability at 7 days of differentiation. As expected, PΔ*cpl* bradyzoites showed a marked loss of viability based on plaque formation (Fig. 8B and C). In contrast, viability of PΔ*cpb* and PΔ*asp1* bradyzoites was no different from that of Pru. Moreover, the decreased viability of PΔ*asp1*Δ*cpl* bradyzoites was no greater than that of PΔ*cpl* bradyzoites compared to that of Pru bradyzoites. Together, these findings suggest that whereas CPL is a key enzyme in bradyzoites, CPB and ASP1 do not play a substantial role in bradyzoite survival.

## DISCUSSION

Toxoplasma gondii cathepsin L (CPL) was previously shown to have a role in protein turnover within the parasite VAC (19, 26). CPL is also necessary for the maturation of T. gondii CPB (23). Extending these findings here, we demonstrate that CPB and CPL colocalize in chronic-stage bradyzoites. Accordingly, it was necessary to assess whether a block in protein turnover in the VAC of CPL-deficient parasites was actually due to a lack of active CPB. Using ingestion assays to assess their contribution to protein digestion in the VAC in tachyzoites, we found that those lacking the *cpb* gene showed the accumulation of host-derived mCherry comparable to that of the Pru parental strain. Similarly, we found comparable punctae in Δ*cpb* and Pru bradyzoites, suggesting that the observed effect of protein accumulation in the VAC of CPL-deficient tachyzoites and bradyzoites is not due to a defect in CPB maturation.

Interestingly, we also found that an aspartyl protease (ASP1), which also occupies the VAC, was not necessary for protein degradation in the VAC or for parasite viability. Further, although ASP1 abundance increases in CPL-deficient parasites, this is apparently due to a lack of its maturation rather than a compensatory mechanism. That parasites lacking both ASP1 and CPL are no worse off than those lacking CPL alone essentially rules out compensation by ASP1 in the absence of CPL. Transcriptomic data sets show that *CPL*, *CPB*, and *ASP1* each are upregulated in chronic-stage bradyzoites (30) compared to acute-stage tachyzoites, which is further supported by our immunoblotting results for CPB and ASP1 in tachyzoites and bradyzoites. Although we found no evidence to suggest CPB and ASP1 are necessary for the turnover of proteins in the VAC in bradyzoites, their increased expression in bradyzoites suggests they have another role in chronic infection. Despite this, we found that neither CPB nor ASP1 is essential for bradyzoite survival. This is in stark contrast to CPL, reinforcing the notion that CPL is the major protease of the VAC in T. gondii bradyzoites.

The nonredundant functions between CPL and other VAC proteases in T. gondii are unlike what has been seen in other eukaryotic organisms previously described. In some other systems cathepsin L is dispensable. For example, in murine models it is only when cathepsins B and L both are deleted from the genome that a strong detrimental phenotype is observed (6, 9).

Cathepsin D is the major aspartyl protease in the mammalian endolysosomal system and is essential for mouse survival (10). This is in contrast to what has been observed in apicomplexans (previously in P. falciparum and reported here in T. gondii), whereby no single aspartyl protease resident within lysosome-like compartments has been shown to be essential to parasite fitness (18). Although not essential for survival, a quadruple knockout of all four FV plasmepsins in P. falciparum resulted in a slower growth rate, delayed schizont maturation, and reduced hemozoin formation (31, 32). This differs from our findings in the related parasite T. gondii, whereby no defect was observed in stage differentiation and protein turnover in Δ*asp1* parasites. It also has been shown here and elsewhere that ASP1 is dispensable for tachyzoite growth (28). Whether ASP1 functions exclusively in the VAC or also plays a secondary role in daughter cell formation associated with the IMC remains unclear. We could not independently validate the association of ASP1 with the IMC in forming daughter cells, because the antibody was insufficiently specific when used to probe replicating parasites despite its specificity for ASP1 in newly invaded parasites (Fig. 4B and Fig. S3E).

Limiting parasite access to essential nutrients by blocking protein turnover has become a major focus of anti-parasite drug development (33–35). In P. falciparum, the hydrolysis of hemoglobin involves the cooperative action of two classes of proteases. In addition to their direct role in protein degradation, falcipains have an indirect role via activation of plasmepsins; however, autoprocessing of plasmepsins has also been observed following falcipain disruption (15). This has implications for the development of inhibitors against P. falciparum proteases involved in protein degradation in the FV, as they will have to block the function of multiple cathepsins simultaneously. Our work reveals a salient aspect of the *Toxoplasma* endolysosomal system in that, unlike *Plasmodium*, there exists an opportunity to target a single nonredundant protease, CPL.

Although our findings suggest noncritical roles for CPB and ASP1 *in vitro*, the contributions of these proteases to infection *in vivo* have been only partly explored. For example, antisense RNA inhibition of CPB expression or treatment with a protease inhibitor significantly decreased T. gondii infection of chicken embryos (36). However, possible off-target effects from antisense inhibition or treatment are difficult to rule out in such studies. Although targeted deletion of ASP1 in the RH strain caused lethal infection of mice with the same kinetics as those for wild-type parasites (28), the high virulence of the RH strain often renders it difficult to measure moderate contributions to infection. Additional studies are necessary to better define the role of CPB and ASP1 during *in vivo* infection.

T. gondii chronically infects one-third of the worldwide population (1). Despite drug development against the infection being an active area of research, there is currently no effective treatment for chronic toxoplasmosis. Although previous work has focused on developing inhibitors against more than one cathepsin protease in T. gondii (37), it is now clear that CPL can be the focus of future efforts targeting disruption of VAC proteolysis. Of particular interest is that the development of compounds that specifically inhibit T. gondii CPL might not have severe side effects resulting from inhibition of host cathepsin L (34).

## MATERIALS AND METHODS

### Parasite cell culture and differentiation into bradyzoites.

Throughout this investigation, human foreskin fibroblast (HFF) cells were used to propagate parasite strains in Dulbecco’s modified Eagle medium (DMEM) supplemented with 10% (vol/vol) cosmic calf serum (CCS) or fetal bovine serum (FBS).

For all bradyzoite conversions, tachyzoites were mechanically lysed by scraping infected HFF monolayers that then were passed through 20- and 23-gauge syringes and a 3-μm filter. Filtered parasites then were counted and allowed to infect fresh monolayers of HFF cells for 24 h. Bradyzoite differentiation was induced using alkaline pH medium and ambient CO_2_ (38–40). Briefly, 24 h after parasites were applied to HFF monolayers, DMEM was replaced with an alkaline differentiation medium (RPMI without NaHCO_3_, 50 mM HEPES, penicillin-streptomycin, 3% FBS, pH 8.25). Differentiation medium was replaced daily.

### Generation of transgenic T. gondii strains.

Strategy, vectors, and repair templates employed to create all the transgenic strains used in this study are described in the supplemental material. The transfection of parasites was performed as described previously (41). Briefly, 2 × 10^7^ tachyzoites were harvested, washed twice in cytomix (120 mM KCl, 0.15 mM CaCl_2_, 10 mM K_2_HPO_4_-KH_2_PO_4_ [pH 7.6], 25 mM HEPES [pH 7.6], 2 mM EGTA, 5 mM MgCl_2_, 2 mM ATP, 5 mM glutathione), and resuspended in 700 μl of cytomix containing either 5 μg of PCR repair template or 50 μg of plasmid. The entire mixture then was transferred to an electroporation cuvette (4-mm gap) and exposed to an electric pulse with an electroporator (BTX 600) set at 2.0 kV and 48 ohms. Electroporated parasites were transferred to fresh HFF monolayers and subjected to drug selection. Clones were isolated by limiting dilution.

### Expression and purification of recombinant proform ASP1.

An ∼1.5-kb DNA fragment encoding amino acids 142 to 610 of the recombinant proform of ASP1 (rproASP1) was PCR amplified from a T. gondii RH cDNA library using Q5 high-fidelity DNA polymerase (NEB) and cloned into a pET22b vector with a 6× His epitope tag fused to the N-terminal end of ASP1. This lacks the first 141 amino acids of ASP1, corresponding to the cytosolic N-terminal domain and transmembrane domain, which could affect protein solubility. The resulting pET22b-6× His-ASP1 expression construct was transformed into the Escherichia coli protein expression strain, ER2566 (NEB), induced with 1 mM isopropyl 1-thio-β-d-galactopyranoside (IPTG) at 37°C for 4 h, and purified using a standard purification procedure as described previously (25). The purified denatured ASP1 protein was refolded in phosphate-buffered saline (PBS) overnight by dialysis. Insoluble protein was removed by centrifugation at 20,000 × *g* for 10 min, and the soluble fraction was concentrated using an Amicon Ultra 30-kDa-cutoff concentrator (Millipore) and stored at –20°C for future use.

### Immunization and affinity purification of anti-ASP1.

Two New Zealand White rabbits were injected with 200 μg of purified recombinant proASP1 in Freund’s complete adjuvant and boosted 3 times at days 14, 21, and 56 with 50 μg of recombinant protein in Freund’s incomplete adjuvant (Cocalico Biologicals, Inc.). The final sera were collected and further affinity purified. Purified recombinant proASP1 was cross-linked to 0.5 ml of AminoLink coupling resin (Thermo Fisher), and affinity-purified anti-ASP1 antibodies were eluted with 0.2 M glycine, pH 3.0. The elution fractions containing the antibodies were pooled, buffer exchanged, and concentrated by using Amicon Ultra 30-kDa concentrators.

### Immunoblotting.

Toxoplasma gondii parasites were purified as described above, counted using a hemocytometer, and pelleted by centrifugation at 1,800 × *g* for 10 min. Parasite pellets were resuspended in SDS-PAGE sample buffer containing 5 mM dithiothreitol and heated at 95°C for 5 min. Parasite proteins were separated by SDS-PAGE on 15% polyacrylamide gels and then transferred to an Immobilon-P polyvinylidene difluoride membrane using semidry electroblotting. Blots were blocked using 0.1% (vol/vol) Tween 20 in PBS (PBST) containing 5% (wt/vol) nonfat dried milk. Blots were probed with the primary antibodies rabbit anti-CPL (1:8,000), mouse anti-CPB (1:2,000), rabbit anti-CPL (1:8,000), affinity-purified rabbit anti-ASP1 (1:2,500), and mouse anti-MIC2 (1:5,000). Secondary horseradish peroxidase-conjugated goat-anti-mouse/rabbit IgG (Jackson Immunoresearch), diluted 1:10,000 in PBST, was applied. Bound antibody was visualized with the chemiluminescence substrate SuperSignal West Pico (Thermo Scientific) and imaged using a G:BOX Chemi XRQ imager (Syngene). Fluorescence immunoblots were as described above, except they were blocked in 50 mM Tris-HCl, pH 7.4, 1.25% (vol/vol) fish gelatin, 150 mM NaCl for 30 min, and secondary antibodies were IRDye 800CW-conjugated goat anti-rabbit IgG conjugated and IRDye 680RD-conjugated goat-anti mouse IgG conjugated. Blots were air-dried before being imaged by a Li-COR Odyssey CLx instrument.

### Immunofluorescence assay.

To confirm protease localization in tachyzoites, indirect immunofluorescence was performed. Parasites were allowed to infect HFF cells for 30 min in 8-well chamber slides at 37°C, 5% CO_2_. Infected cells then were fixed using 4% (vol/vol) methanol-free formaldehyde in PBS. Cells were permeabilized with 0.1% (vol/vol) Triton X-100 for 10 min, followed by 20 min of blocking with PBS containing 10% FBS. Blocked monolayers were incubated at room temperature (RT) for 40 min with the primary antibody mouse anti-CPB (1:200), rabbit anti-CPL (1:500), or affinity-purified rabbit anti-ASP1 (1:200). Following washes in PBS–1% FBS–1% normal goat serum, slides were probed with secondary fluorophore-conjugated antibodies (Jackson Immunoresearch), mounted using Mowiol, and imaged on a Zeiss Axio inverted fluorescence microscope equipped with a Zeiss AxioCam 305 scMOS digital camera.

For indirect immunofluorescence on bradyzoites, freshly lysed parasites were placed onto an HFF cell monolayer and left at 37°C and 5% CO_2_ overnight. Invaded parasites were subsequently stimulated to differentiate into bradyzoites by culturing in alkaline differentiation media and incubating at 37°C and 0% CO_2_ for 7 days. Bradyzoite cultures were fixed, probed with antibodies, and mounted as described above.

### Parasite ingestion assay.

To assess the degradation of proteinaceous material within the lysosome-like VAC in tachyzoites, the previously described ingestion assay was used (42). Briefly, modified CHO-K1 cells were induced to express cytosolic mCherry following treatment with doxycycline (2 μg/ml) for 5 days prior to infection. On the sixth day, freshly harvested parasites were allowed to infect and ingest cytosolic mCherry from doxycycline-treated CHO-K1 cells for 4 h. After incubating, parasites were harvested from these cells. Purified parasites were treated at 12°C for 1 h with freshly prepared 1 mg/ml pronase (Roche)–0.01% saponin–PBS before washing, placing them on slides precoated with Cell-Tak (Corning), and fixing with 4% formaldehyde in PBS (20 min at RT). Fixed parasites were washed three times with PBS and permeabilized by incubation with PBS with 0.1% Triton X-100 for 10 min at RT. Slides then were mounted with Mowiol and imaged as described above. Samples were coded and enumerated blindly.

### Tachyzoite growth assays.

To determine the contributions of CPB, CPL, and ASP1 to intracellular tachyzoite growth, the relative growth rate of Pru and each knockout strain was compared based on the number of parasites per parasitophorous vacuole 24 and 48 h postinvasion. Freshly harvested tachyzoites were placed onto a monolayer of confluent HFF cells grown on coverslips in a 6-well plate containing DMEM supplemented with 10% CCS. Parasites were allowed to invade for 1 h, at which point parasites that had not invaded were washed away with DMEM supplemented with 10% CCS. Parasites were left to grow for a further 24 and 48 h before washing with PBS three times and fixing with 4% (vol/vol) methanol-free formaldehyde in PBS. Cells were permeabilized, blocked, and washed as described above. To visualize individual parasites for counting, the prepared slides were incubated for 40 min at RT with rat anti-SAG1 antibodies, which were generated by a commercial service (Pierce) using baculovirus-derived recombinant SAG1 (kindly provided by Martin Boulanger), followed by secondary Alexa-594-conjugated anti-rat antibody (Jackson Immunoresearch). The number of parasites per parasitophorous vacuole was counted for each strain.

### Puncta assays.

To assess the formation of puncta (accumulation of dense material) in each strain, we followed the puncta quantification assay described previously (26). Briefly, HFF cell monolayers on coverslips were infected with T. gondii tachyzoites and differentiated to bradyzoites over the course of 7 days (as described above). For direct immunofluorescence of bradyzoite cyst walls, fluorescein-labeled Dolichos biflorus agglutinin (DBA; Vector Labs) was diluted 1:400 and incubated with infected monolayers that had been fixed and made permeable (as described above). The fluorescent signal derived from DBA staining allows automatic detection of the bradyzoite-containing cyst area using ImageJ software. Cyst images were captured with a 100× oil objective as described above. Automatic bradyzoite cyst identification and puncta quantification were performed in ImageJ using the following parameters, which were described previously (26). Maximum entropy thresholding on the GFP channel was used to identify cysts. This was followed by the identification of objects with areas between 130 and 1,900 μm^2^. Particles (punctae) under the GFP mask (and, therefore, within a cyst) were analyzed by automatic local thresholding on the phase image using the Phansalkar method, with the following parameters: radius, 5.00 μm; *k* value, 0.5; *r* value, 0.5. Puncta were measured from the resulting binary mask by particle analysis according to the following parameters: size, 0.3 to 5.00 μm; circularity, 0.50 to 1.00.

### Bradyzoite differentiation assay.

Immunofluorescence assays were performed to determine the relative rate at which parasites differentiated from the tachyzoite stage to the bradyzoite stage, based on the amount of tachyzoite-specific SAG1 and bradyzoite-specific GFP present within parasitophorous vacuoles/developing cysts. Freshly lysed tachyzoites were placed onto confluent HFF monolayers on coverslips (in 6-well plates) and left to incubate at 37°C and 5% CO_2_ for 24 h. Following this, the medium was removed from each well and alkaline medium was added, along with placement in incubators lacking CO_2_ to induce bradyzoite conversion (still maintained at 37°C). Coverslips were fixed and stained with the tachyzoite-specific marker rat anti-SAG1 (1:1,000) following 1, 2, 3, and 4 days of differentiation. Strains used in this study express GFP under the bradyzoite-specific LDH2 promoter, and this is used as a marker for bradyzoites. Cysts that displayed 50% or more staining by either rat anti-SAG1 antibodies or GFP were considered positive.

### Bradyzoite viability assays.

Bradyzoite viability was assessed by combining plaque assay and quantitative PCR (qPCR) analysis of genome number, as previously described (26). Briefly, HFF cell monolayers were infected with T. gondii tachyzoites in 6-well plates. Following host cell invasion, tachyzoites underwent differentiation to bradyzoites as described above, resulting in the generation of *in vitro* tissue cysts. Differentiation was carried out over the course of 7 days, replacing the alkaline medium daily. Following differentiation, the culture medium in each well was replaced with 2 ml Hanks balanced salt solution, and cysts were liberated from the infected HFF monolayers by mechanical extrusion by lifting cells with a cell scraper and syringing several times through 25-gauge needles. In the next step, 2 ml of prewarmed 2× pepsin solution (0.026% pepsin in 170 mM NaCl and 60 mM HCl, final concentration) was added to each sample, and samples were left to incubate at 37°C for 30 min. Reactions were stopped by adding 94 mM Na_2_CO_3_, removing the supernatant after centrifugation at 1,500 × *g* for 10 min at RT, and resuspending pepsin-treated parasites in 1 ml of DMEM without serum. Parasites were enumerated, and 1,000 parasites per well were added to 6-well plates containing confluent monolayers of HFFs in D10 medium in triplicate. To allow for the formation of bradyzoite plaques, parasites were left to grow undisturbed for 10 days. After this period, the number of plaques in each well was determined by counting plaques with the use of a light microscope. Five hundred microliters of the initial 1 ml of pepsin-treated parasites was used for genomic DNA purification, performed using the DNeasy blood and tissue kit (Qiagen). Genomic DNA was eluted in a final volume of 200 μl. To determine the number of parasite genomes per microliter, 10 μl of each genomic DNA sample was analyzed by qPCR in duplicate using the tubulin primers TUB2.RT.F and TUB2.RT.R (42). Quantitative PCR was performed using Brilliant II SYBR green qPCR master mix (Agilent) and a Stratagene Mx3000PQ-PCR machine. The number of plaques that formed per genome then was calculated. For each experimental replicate, the plaques/genome was normalized to the parental control, and this normalized value was used for statistical analysis.
