# Supplementary figures and images for: Toxoplasma Cathepsin Protease B and Aspartyl Protease 1 Are Dispensable for Endolysosomal Protein Digestion
Source: mSphere. 2020 Feb 12;5(1):e00869-19. doi: 10.1128/mSphere.00869-19 (PMC7021471; doi:10.1128/mSphere.00869-19)

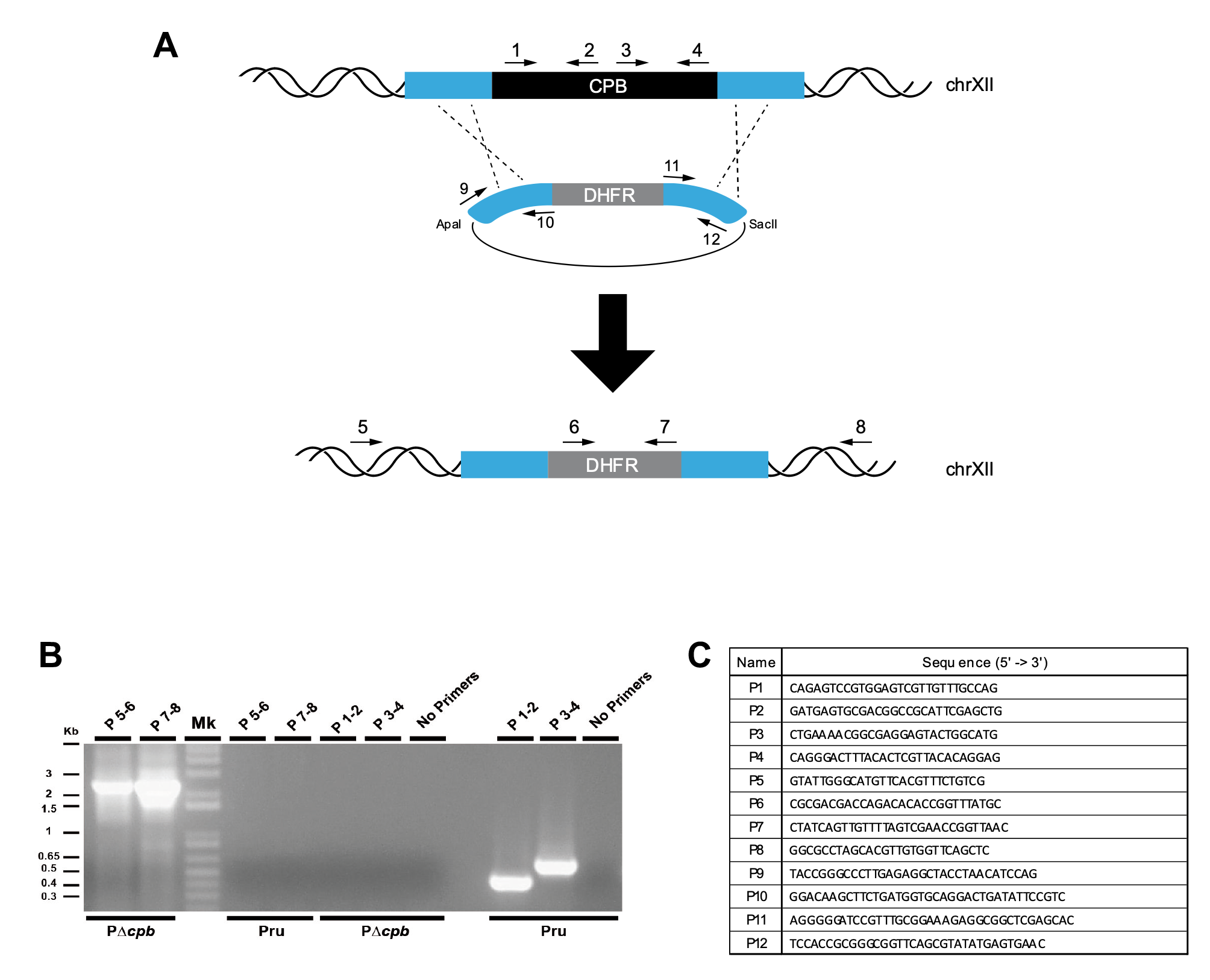

Supplement: FIG S1 [file mSphere.00869-19-sf001.tif]

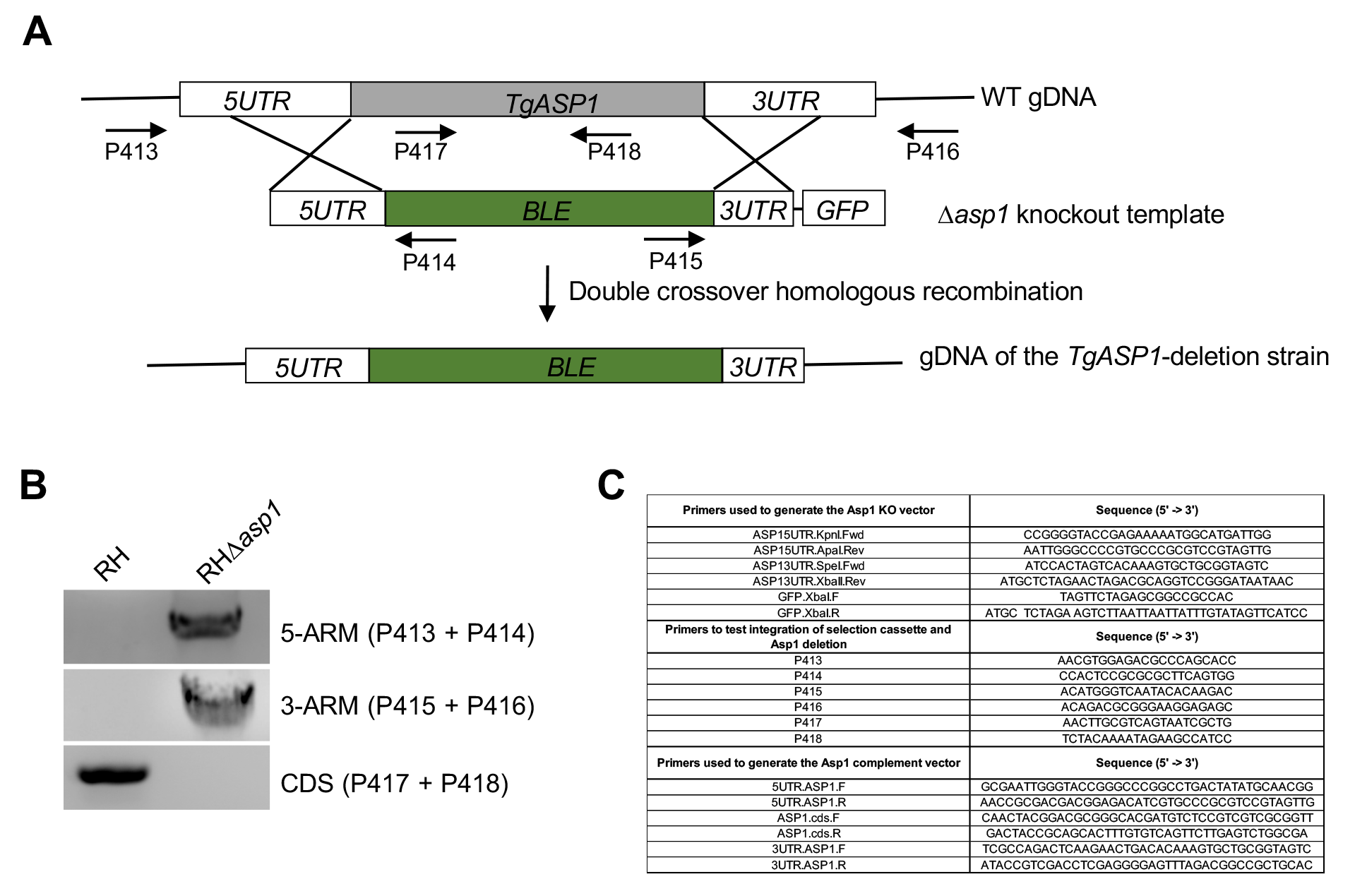

Supplement: FIG S2 [file mSphere.00869-19-sf002.tif]

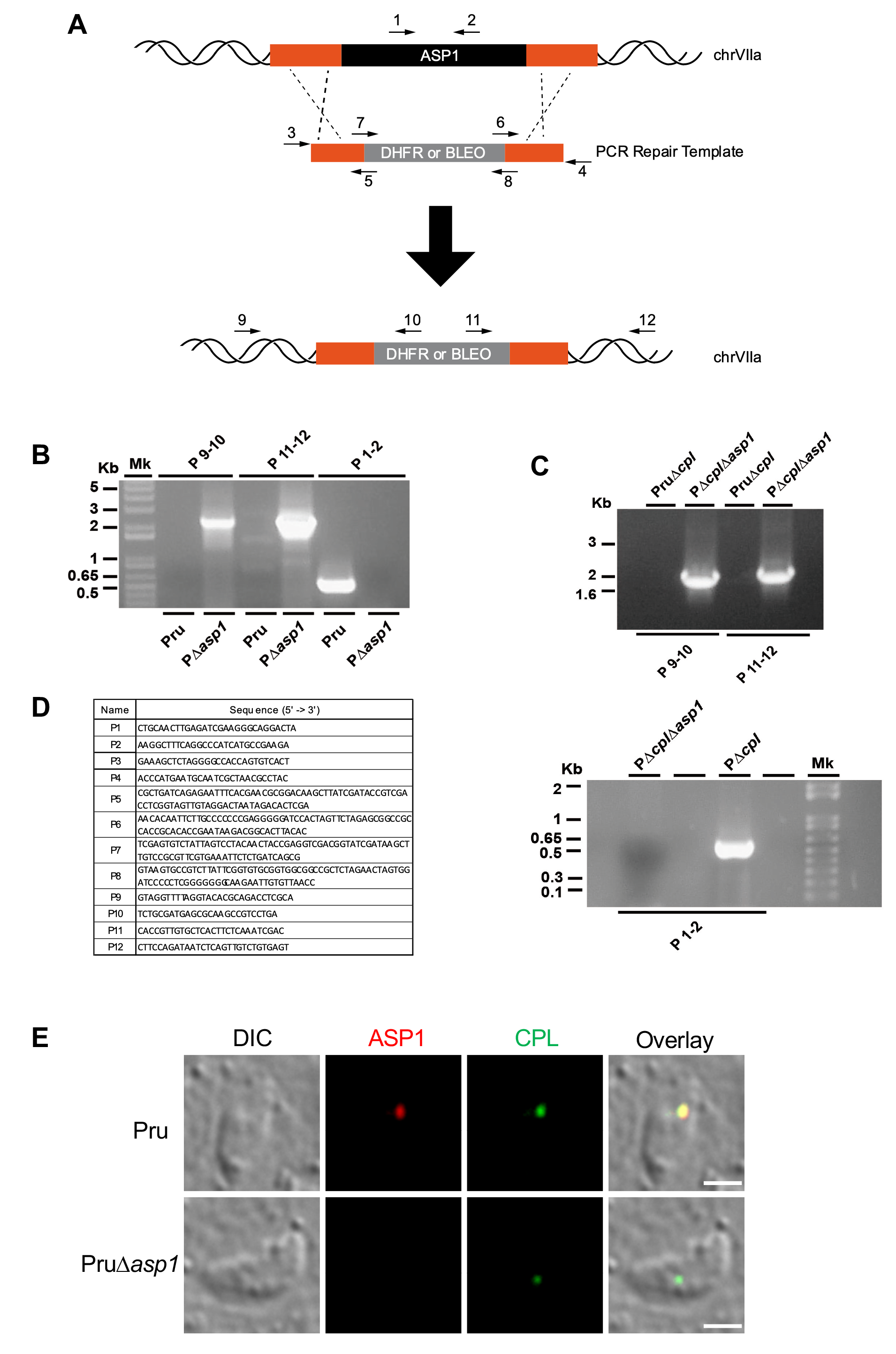

Supplement: FIG S3 [file mSphere.00869-19-sf003.tif]
